# Effect of doping of KDP crystal with amino acid l-arginine on the strength properties and character of laser damage

**DOI:** 10.1016/j.jcrysgro.2017.08.010

**Published:** 2017-11-15

**Authors:** E.F. Dolzhenkova, E.I. Kostenyukova, O.N. Bezkrovnaya, I.M. Pritula

**Affiliations:** Institute for Single Crystals, NASU, 60 Nauki Ave., 61001 Kharkov, Ukraine

**Keywords:** A. Optical materials, B. Crystal growth, B. Mechanical properties, D. Crystal structure, D. Fracture

## Abstract

Studied were the strength characteristics of KDP crystals doped with l-arginine under a concentrated load and irradiation of the first harmonic YAG:Nd^3+^ laser. The crystals were obtained by means of the temperature reduction method on a point seed, the content of l-arginine in the aqueous solution being 0.3, 0.4, 1.0 and 1.4 wt%. The character of the dependence of KDP microhardness versus the concentration of amino acid in the crystal was investigated. The regularities of brittle damage of the doped KDP crystal at mechanical testing and laser irradiation were shown to be similar. As confirmed in the study, the planes of easy crack extension in the crystal are {2 2 1}, (1 0 0), and (0 0 1) planes, the cracks mainly propagate parallel to {2 2 1} planes. The mechanical and laser strength values of doped KDP crystals were evaluated.

## Highlights

• Mechanical and laser strength values of KDP:l-arginine crystals were determined.
• Pure KDP and KDP:l-arginine crystals demonstrate “reverse indentation size effect”.
• Peculiarities of mechanical and laser damage of doped KDP crystal are similar.

## Graphical abstract

**Figure:**
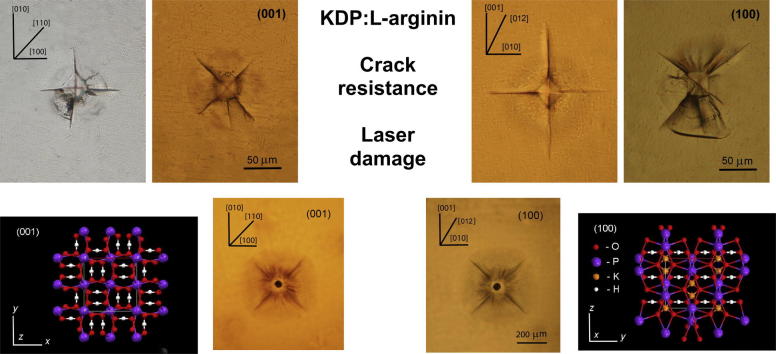

## 1 Introduction

KDP (KH_2_PO_4_) group crystals which belong to a vast class of electrooptical and nonlinear optical media have found wide practical use in modern nonlinear optics and optoelectronics [1]. These crystals are applied in high-power laser systems with a peak power exceeding >1 GW/cm^2^ emitting pulses with an energy of several kJ. This is due to a unique set of physical properties of group KDP crystals, in particular, their high transparency in a wide (ranging from the UV to near IR) spectral region, high laser damage resistance, good optical homogeneity combined with optical nonlinearity optimal for powerful high-energy laser systems. Moreover, the existing technologies make it possible to grow and mechanically treat large-size KDP crystals [2]. One of the methods for raising the efficiency of second harmonic generation (SHG) is introduction of organic molecules, e.g. amino acids, which possess high nonlinear coefficients, into the matrix of KDP crystal. As shown in [3], the efficiency of SHG for KDP with 0.3 wt% of l-arginine (l-arg) and KDP with 0.4 wt% of l-arg rises by a factor of 1.33 and 1.74, respectively, in comparison with that of pure KDP. On the other hand, in a number of studies [4], [5] it has been revealed that doping with l-arg and other amino acids leads to certain decrease of the values of microhardness for KDP. It is well-known [6] that laser damage of KDP crystals essentially depends on their strength characteristics, and is followed by the appearance and propagation of micro-cracks caused by mechanical or heating pulse in the process of irradiation. Despite good optical properties, high structure perfection and the existence of the reproducible growth technology, the real laser-induced damage threshold in KDP crystals is essentially lower than the theoretically possible one [2]. Damage of the material under the action of laser pulses is one of the factors limiting the use of the crystals for generation of higher harmonics. Only few works [e.g. [4], [5], [6], [7], [8] are devoted to the regularities of brittle fracture in pure KDP crystals subjected to mechanical loading and laser irradiation. Nowadays, information about the damage processes in KDP doped with l-arg and other amino acids is absent. The present work reports the study of the strength properties of KDP crystals containing l-arg additions.

## 2 Material and investigation technique

KDP crystals, both pure and the doped, were grown from aqueous solution by the method of temperature reduction onto a point seed measuring 5 × 5 × 10 mm^3^. The content of l-arg in the mother solution (pH 4.0 ± 0.1) was 0.3, 0.4, 1.0 and 1.4 wt%. The solutions were filtered and then overheated during 24 h at T = 80 °C. The relative solution supersaturation σ was ∼2%, the rate of temperature lowering being 0.3° per day. To provide dynamical crystal growth conditions, the solution contained in the crystallizer was mixed at a rate of 70 rpm. The average crystal growth rates along a growth direction were V_z_ = 2.0 mm per day and V_x,y_ = 1.3 mm per day. The dimensions of KDP and KDP:l-arg (1.4 wt%) crystals were 79 × 65 × 54 mm^3^ and 52 × 42 × 110 mm^3^, respectively.

**Fig. 1:**
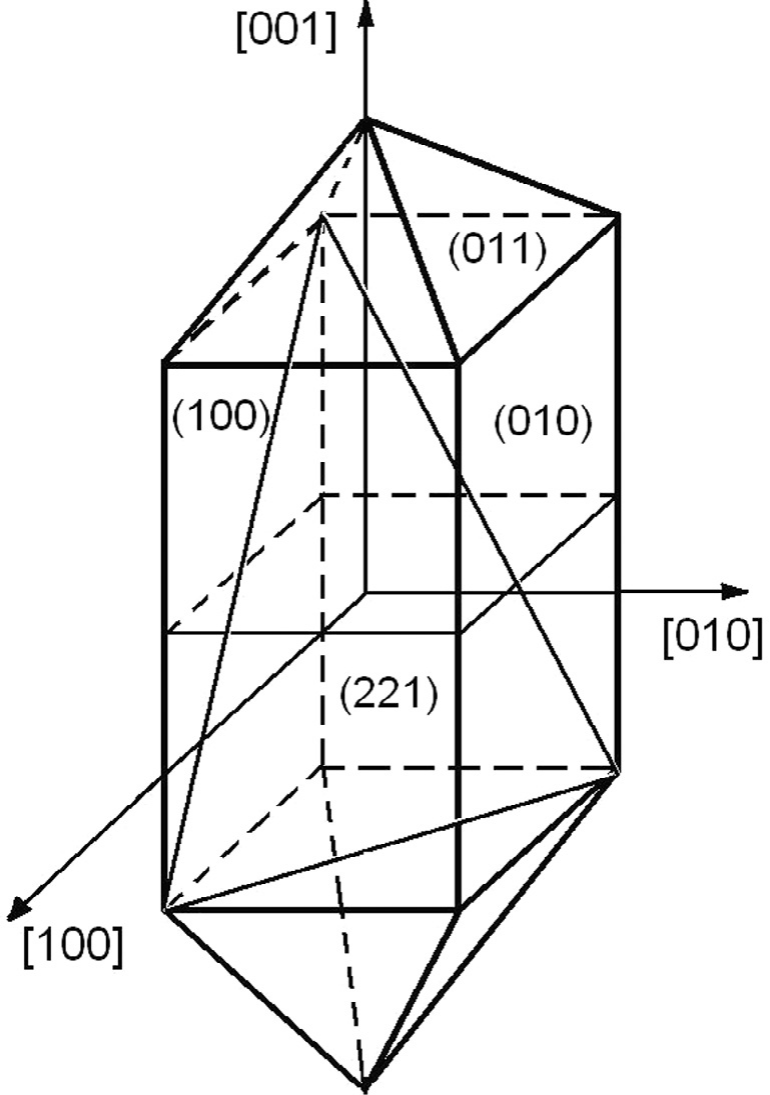
Morphology of KDP crystal faces.

At room temperature the structure of potassium dihydrogen phosphate is characterized by the space group $I\overset{‾}{4}2d$ [9]. The morphology of KDP crystal is formed by tetragonal prism {1 0 0} and tetragonal bipyramid {1 0 1} faces (Fig. 1). All the grown crystals had well-developed prismatic and pyramidal growth sectors. The samples to be investigated were obtained from both growth sectors. We have previously shown that the doping in the range of 0.3–1.4 wt% concentrations lead to an increase of the efficiency of second harmonic generation by 1.33–2.53 times in the pyramidal sector, and in the 2.36–3.95 times in prismatic sector [10], [11].

The study was performed on the mechanically ground and polished faces (0 0 1) and (1 0 0). After mechanical treatment the surface roughness was measured by Mod. TR_200 profilometer_profilograph. For each sample, the surface roughness average parameter *Ra* was determined by the results of four to five measurements. Standard deviation was 6.3% for the surface roughness parameter. The surface micro relief height of the crystals was 15 nm.

The mechanical properties of the crystals were investigated by the method of microhardness based on deformation by concentrated load. Indentation was realized using a PMT-3 device with the standard tetrahedral indenter, the loads ranged from 0.2 to 2 N. Most indentations were oriented along the diagonals of the square impression parallel to the [1 0 0] and [0 1 0] directions on the plane (0 0 1), and parallel to the [0 1 0] and [0 0 1] directions on the plane (1 0 0). The obtained indenter imprint and the radial cracks around it were measured by means of a computer-aided optical microscope Zeiss Axioskop 40A POL. The microhardness value was determined from the relation $H_{V}=1854P_{1}/a^{2}$ (kgf/mm^2^), where *P*_1_ is the indenter load (g), *a* is the imprint diagonal (μm). The crack resistance was estimated from the value of the threshold stress intensity factor *К*_C_ for fracture according to the formula: $K_{C}=0.016\cdot {\left(\frac{E_{1}}{H_{V}}\right)}^{1/2}\cdot \frac{P_{1}}{l^{3/2}}$ (MPa·m^1/2^) [12], where *l* is the radial crack length from indentation centre to crack tip, *E*_1_, the Young’ modulus (*E*_1_ = 38.7 GPa) [8]. The measurements were realized on 10 imprints at each designated indentation load. The standard deviation in the measured value of mean microhardness was about 2%, whereas this deviation in the average crack length was found to be up to 10%.

The samples were also irradiated by a single-mode pulsed YAG Nd^3+^ laser at the wavelength λ = 1.064 μm according to the scheme n – 1 – n (90 pulses with the same energy, the samples were moved in the plane perpendicular to the laser beam), the radiation energy was 2.75 μJ, the pulse repetition frequency and pulse duration being 1 Hz and 10 ns, respectively. The criterion of laser breakdown was visually observed spark of high-temperature glow at the crystal damage. The measurement error was 10%.

## 3 Experimental results

**Fig. 2:**
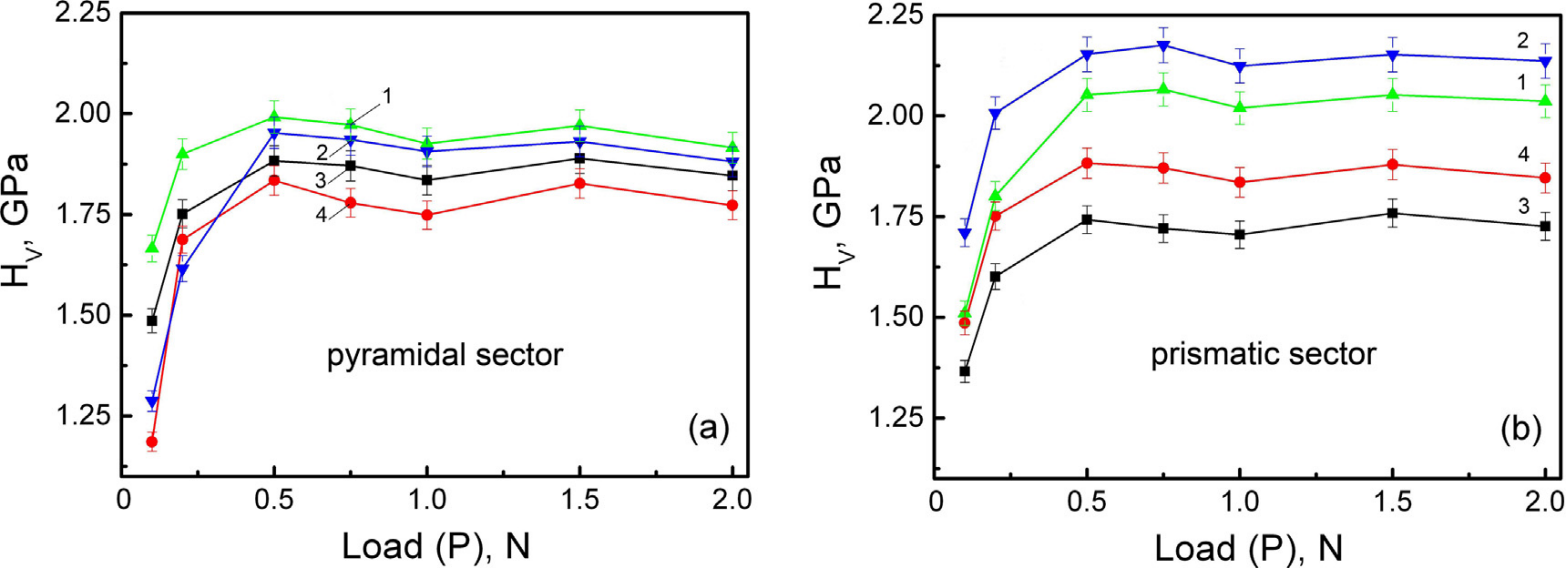
Loading curves for the pyramidal (a) and prismatic sectors (b) in the planes (1 0 0) (1) and (0 0 1) (2) of pure KDP, and in the planes (1 0 0) (3) and (0 0 1) (4) for KDP:l-arg crystal.

Fig. 2 shows the loading curves for the faces (0 0 1) and (1 0 0) for pure KDP and KDP:l-arg crystals. The microhardness of both crystals rises with the load and is described by a curve with a small maximum. The error bars in the figure are the standard deviations in the measured *H*_v_. Fig. 2 is an example of “reverse indentation size effect” in which microhardness increases with the rise of indentation load.

At loads of 0.5–2 N the microhardness of the planes (1 0 0) and (0 0 1) in the pyramidal sector of pure KDP crystal is practically the same. On the average, the microhardness of the samples cut out from the prismatic sector is somewhat higher than those obtained from the prismatic sector; thereat the hardness number for the plane (0 0 1) exceeds that for the plane (1 0 0) by ∼6–7%.

Doping of KDP with 1.4 wt% l-arg resulted in a diminution of the crystal’s load-independent microhardness by ∼5–9% (this difference is not easily recognized) for the pyramidal sector and by ∼14–18% for the prismatic sector. The hardness number in the pyramidal sector for the face (1 0 0) of the doped crystal exceeded the values obtained for the face (0 0 1) by ∼6%. In the prismatic sector the hardness anisotropy of the planes (1 0 0) and (0 0 1) of the crystal containing the amino acid remained unchanged.

**Fig. 3:**
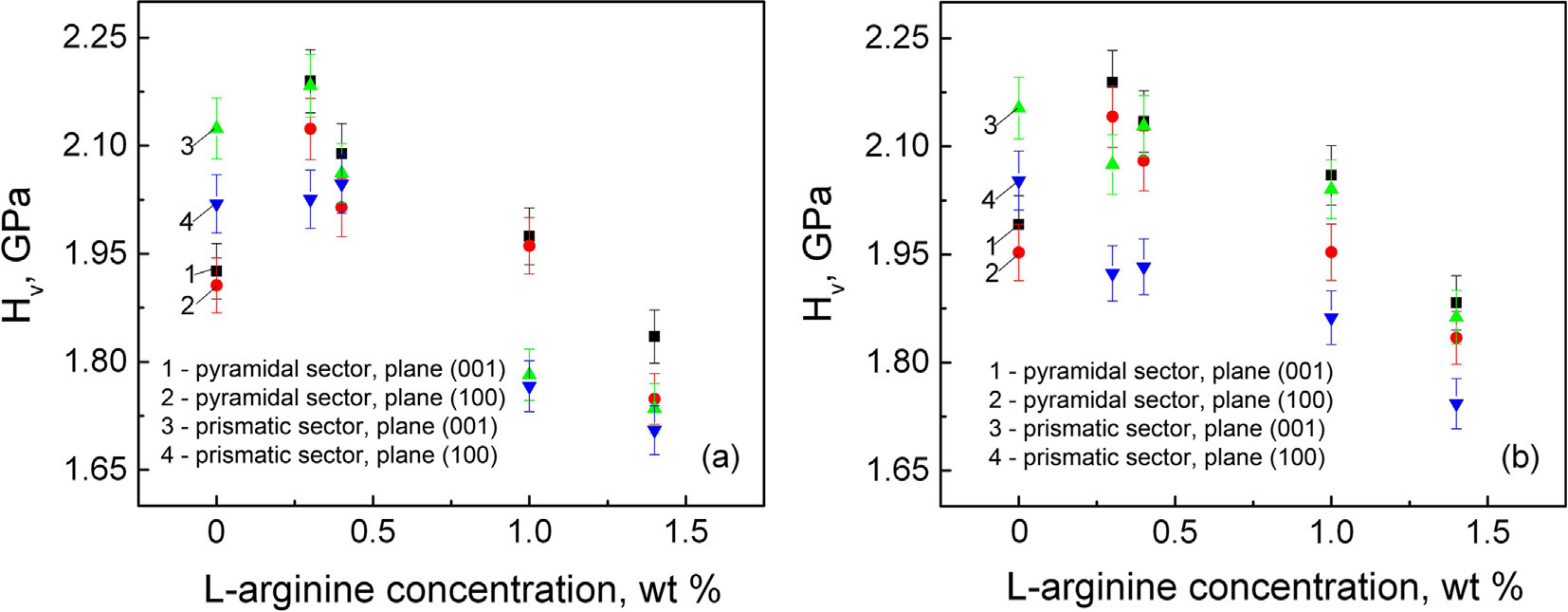
Microhardness of KDP crystal versus the content of l-arg in the solution at the indenter loads: *P* = 1.0 N (a), *P* = 0.5 N (b).

The character of the dependence of the microhardness on the concentration of l-arg for the pyramidal and prismatic sectors of KDP crystal is defined by the indenter loading. In particular, the experiments performed at an indenter load of 1 N have shown that doping with 0.3 and 0.4 wt% of l-arg leads to certain hardening of the crystal (Fig. 3-a). When the indenter load is 0.5 N the concentration dependence shows the hardness maximum only for the samples cut out from the pyramidal sector (Fig. 3-b). In the samples obtained from the prismatic sector the hardness uniformly diminishes with the rise of the content of the amino acid in the crystal.

**Fig. 4:**
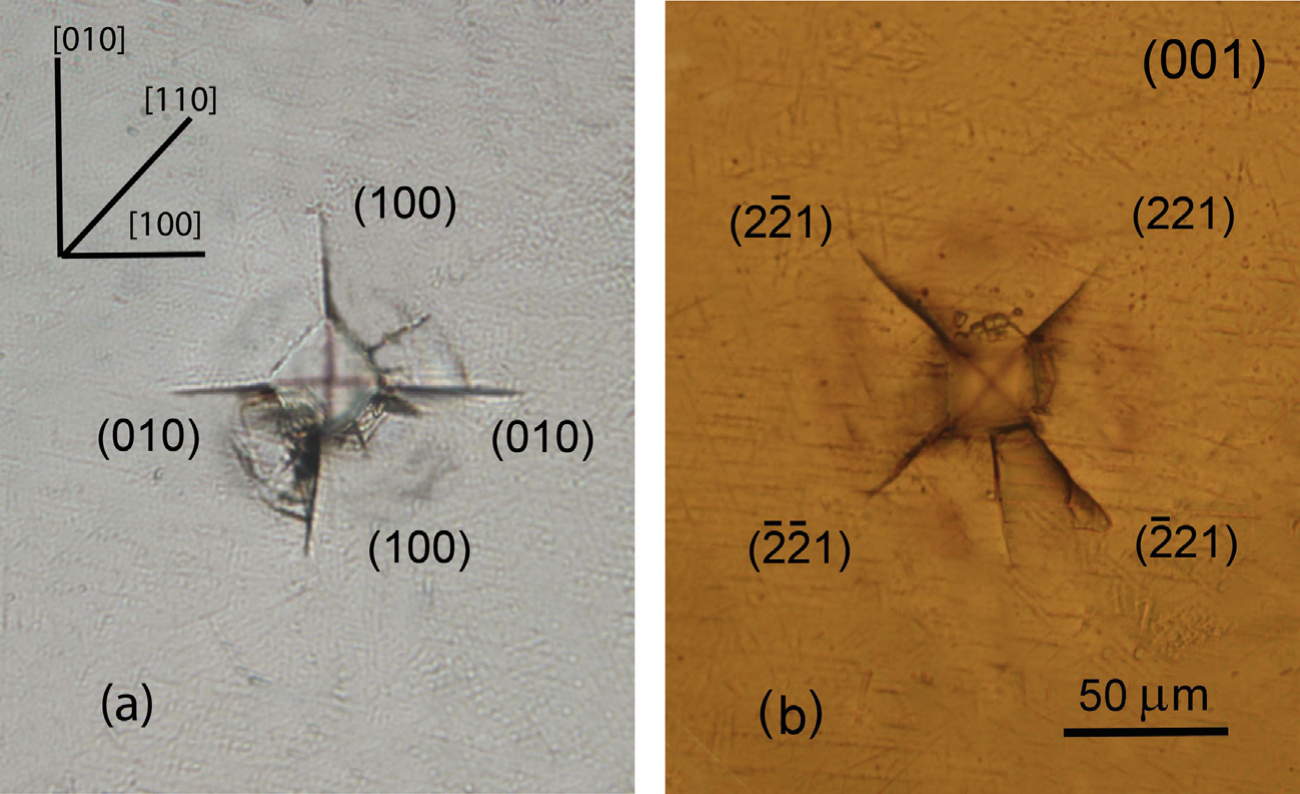
Micrographs of cracks near the indent (P = 1 N) on the surface (0 0 1) of KDP:l-arg (1.4 wt%) crystal. The indenter diagonals are parallel to the directions [1 0 0] (a) and [1 1 0] (b).

**Fig. 5:**
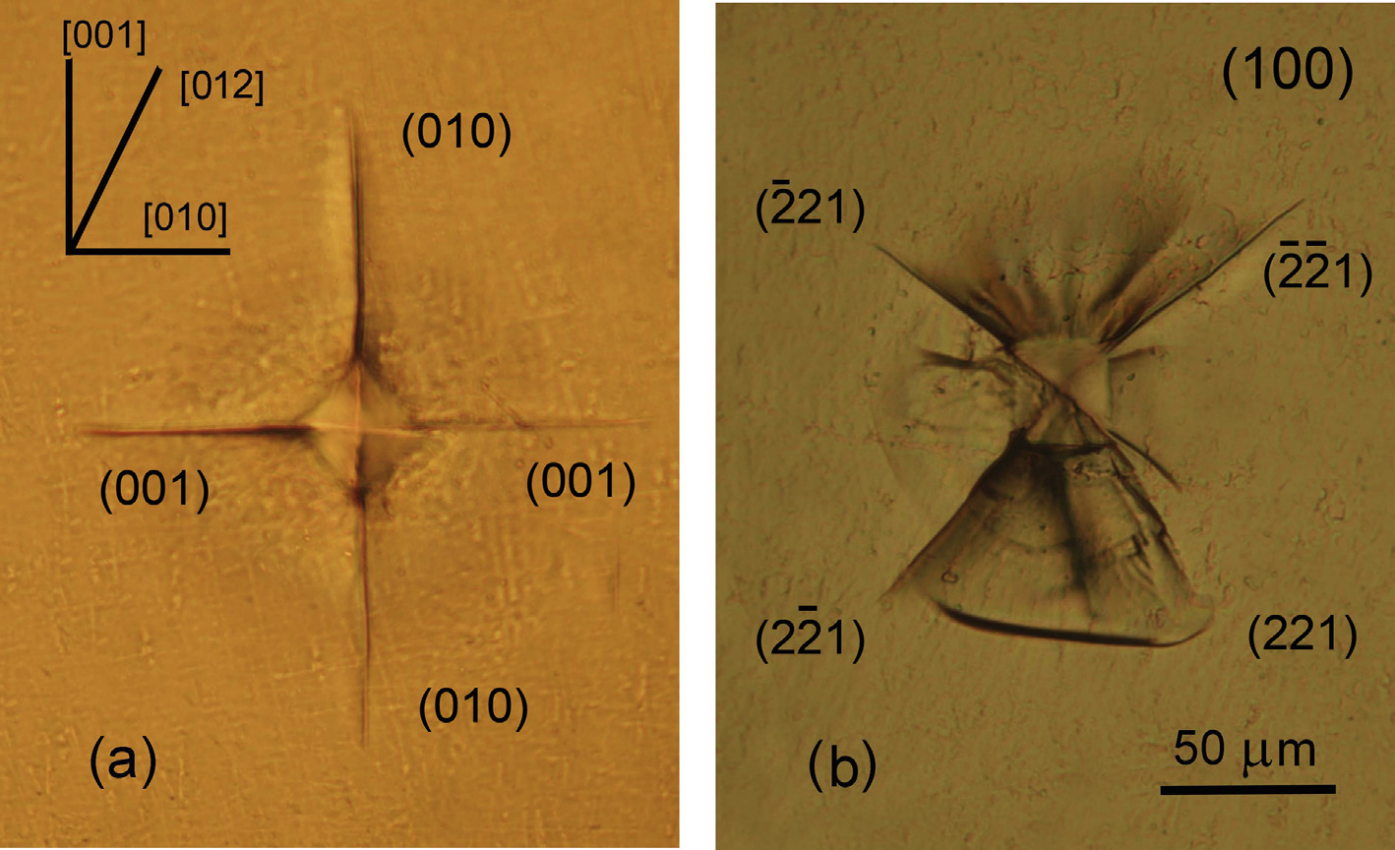
Micrographs of cracks near the indent (P = 1 N) on the surface (1 0 0) of KDP:l-arg (1.4 wt%) crystal. The indenter diagonals are parallel to the directions [0 1 0] (a) and [0 1 1] (b).

**Fig. 6:**
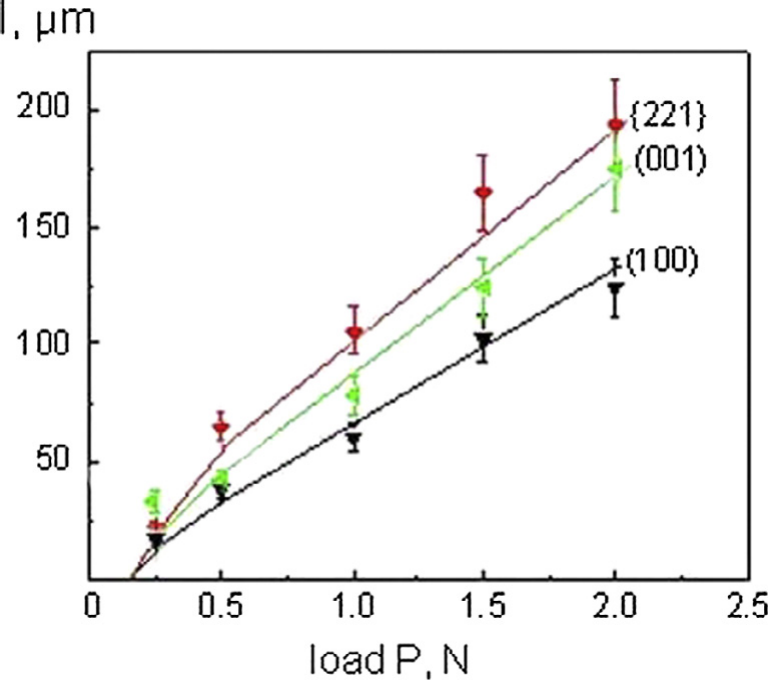
Half-cracks size of cracks propagated in KDP:l-arg (1.4 wt%) crystal along planes of easy crack extension {2 2 1}, (0 0 1), (1 0 0) versus the indentor load.

Presented in Fig. 4, Fig. 5 is the image of the indenter imprint obtained at an indenter load of 1 N on the planes (0 0 1) and (1 0 0) of KDP:l-arg (1.4 wt%) crystals cut out from the prismatic sector. During indentation of the plane (0 0 1) (Fig. 4) the direction of the cracks coincides with the direction of the imprint diagonals when the latter are parallel to the directions [1 0 0] and [1 1 0]. As seen during the examination in transmitted light, the radial cracks formed in the process of indentation, propagate in the planes (1 0 0) – perpendicular to the examined surface and {2 2 1} – inclined to it. When the plane (0 0 1) was indented, the cracks around the indenter imprint were propagated along the [1 0 0], [0 1 0] and 〈1 1 0〉 directions corresponding to the traces of the planes (0 1 0), (1 0 0) and {2 2 1}, respectively. Besides the radial cracks, a developed system of lateral cracks parallel to the indented plane (0 0 1) is observed around the imprint. The indentation pattern for the face (1 0 0) (Fig. 5) shows that the radial cracks formed around the imprint propagate in the directions [0 0 1], [0 1 0] and [2 1 0]. While studying the zone of brittle fracture in transmitted light there has been revealed that the cracks develop in the plane {2 2 1} inclined to the examined surface and to (0 0 1) perpendicular to the latter. The cracks which were parallel to the [0 0 1], [0 1 0] directions correspond to the intersections of the planes (0 1 0) and (0 0 1) with the indented plane (1 0 0). The inclined planes {2 2 1} crosses the plane (1 0 0) along the 〈0 1 2〉 directions. Fig. 6 illustrates the dependence of the crack length *l* on the indentation load *P* applied on these investigated planes. At the same value of *P* the cracks propagate rather along the plane {2 2 1} than along (1 0 0) and (0 0 1).

Presented below are the values of the fracture toughness in the planes of easy crack propagation (Table 1). One can see that the changes in the crack resistance of the doped crystals (decreasing by 1.1–1.5 times within the measurement error) correspond to the changes in their microhardness. Thereat, the anisotropy of the resistance to crack propagation exceeded the hardness anisotropy. The lowest values of the fracture toughness were observed in the plane {2 2 1} both in pure and doped crystals. The crack resistance of the samples containing 0.3 and 0.4 wt% of l-arg does not differ from the one of the pure crystals.

**Table 1:** Crack resistance of pure and doped KDP crystals.

Plane	{2 2 1}	(1 0 0)	(0 0 1)
*K*_c_, MPa·m^1/2^ KDP pure	0.22 0.26	0.33 0.34	0.38* 0.36**
*K*_c_, MPa·m^1/2^ KDP:l-arg (1.4 wt%)	0.17 0.18	0.26 0.27	0.30 0.29

* For the prismatic sector.

** For the pyramidal sector.

**Fig. 7:**
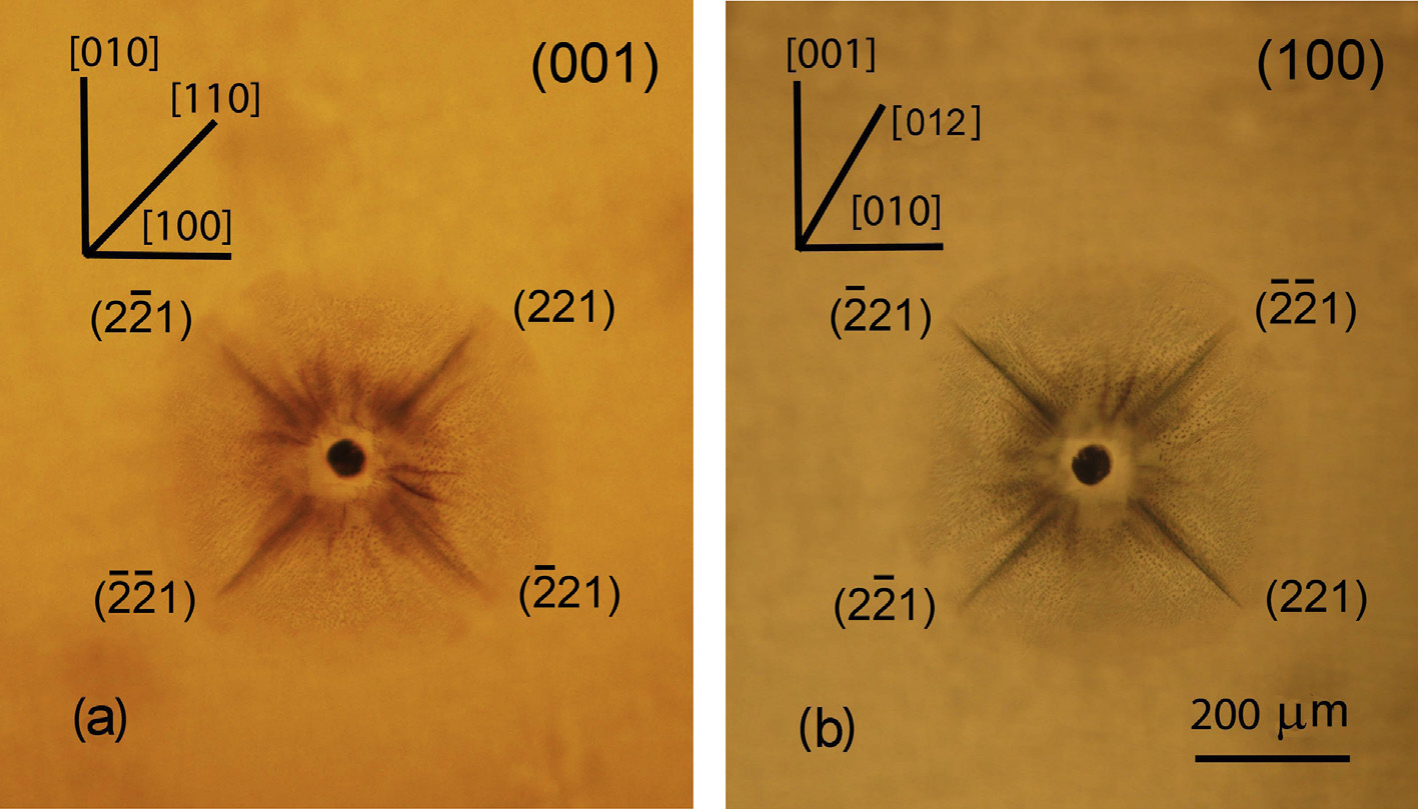
Micrographs of the damage tracks in KDP:l-arg (1.4 wt%) crystal observed from the planes (0 0 1) (a) and (1 0 0) (b).

The cracks around damage spot pattern in KDP:l-arg (1.4 wt%) caused by laser pulse viewed perpendicular to the planes (0 0 1) and (1 0 0), are shown in Fig. 7. Laser induced damage of the crystal KDP also occurs parallel to the planes of {2 2 1}, (1 0 0) and (0 0 1) type. The damage tracks propagated parallel 〈1 1 0〉, which were obtained with the laser beam directed along the axis [0 0 1], correspond to the intersection of the planes {2 2 1} with the plane (0 0 1) (Fig. 7-a). The damage tracks stretched along 〈0 1 2〉, observed from the plane (1 0 0) coincide with the crosses the investigated plane (1 0 0) and the planes {2 2 1} (Fig. 7-b). However, in the case when the damage occurs at local heating at the laser focus point, the anisotropy of crack propagation is much more essential than the one at local application of mechanical load. The planes of predominant crack propagation is {2 2 1}, the probability of laser-induced damage along the planes (1 0 0) and (0 0 1) is considerably lower.

Table 2 presents the average values of laser-induced damage threshold in the pure and doped crystals for the pyramidal and prismatic growth sectors measured in the laser irradiation directions [0 0 1] and [1 0 0]. The value of laser breakdown threshold of KDP:l-arg (1.4 wt%) obtained for the prismatic sector is lower than the one of pure KDP crystal, although they are within the experimental error. However, for the pyramidal sector these values are higher. In pure crystals laser strength in prismatic growth sector is somewhat higher than in pyramidal one; in doped crystals an inverse picture is observed. The laser damage threshold of KDP crystals is limited by the presence of impurities. The higher crystalline perfection of pyramidal growth sector may be responsible for larger laser damage threshold in the said sector in doped crystals. For the plane (0 0 1) the laser-induced damage threshold for pure and doped KDP crystals is higher than that for the plane (1 0 0). This agrees with their lower microhardness values in the plane (1 0 0) in comparison with the one in the plane (0 0 1) in both growth sectors.

**Table 2:** Laser damage threshold of pure and KDP:l-arg (1.4 wt% l-arg).

Crystals	Prismatic growth sector		Pyramidal growth sector	
	irradiation direction [0 0 1], J/cm^2^	Irradiation direction [1 0 0], J/cm^2^	Irradiation direction [0 0 1], J/cm^2^	Irradiation direction [1 0 0], J/cm^2^
KDP pure	42.3	37.29	41.98	28.15
KDP:l-arg (1.4 wt%)	40.52	32.15	56.09	42.73

## 4 Discussion

The dependence of microhardness *H*_v_ on indentation load - “reverse indentation size effect” (Fig. 2) in [13] is attributed to the influence of the surface layer of the crystal which is the most essential at low indenter loads. At low loads the thickness of the surface-adjacent deformed layer resulting from machining is comparable with the indenter penetration depth, so the imprint undergoes the action of tensile surface stresses which increases its size. As the penetration depth increases, the effect of the distorted zone adjoining the crystal surface on the hardness becomes less noticeable. At higher loads (0.5–2 N) when these stresses are overcome, and the indentor penetrates the undistorted material, the microhardness value is independent of the load.

Hardness anisotropy of (0 0 1) and (1 0 0) planes, and difference in microhardness values of the samples cut from the pyramidal and prismatic sectors of pure KTP crystals may be connected with predominant incorporation of the metal ions contained in the solution, such as Fe^3+^, Cr^3+^, Al^3+^ etc. into the prismatic growth sector [14]. The charge state of the tetragonal prism {1 0 0} and tetragonal bipyramid {1 0 1} surfaces are different. The faces of the tetragonal bipyramid consist of two layers of K^+^-cations and two layers of H_2_PO_4_^−^ -anions. The positively charged ions K^+^ define on the surface of the tetragonal bipyramid, the faces {1 0 1} are positively charged. The surface of the tetragonal prism is formed by phosphate-ions mixed with K^+^-ions, therefore they are electrically neutral [15]. In the process of KDP crystal growth the faces {1 0 1} adsorb different anions and reject cations, {1 0 0} faces capture both positive and negative impurities. The impurity ions impede dislocation motion and to some extent favor the rise of the crystal’s hardness in the prismatic growth sector. Deformation of the surface (1 0 0) involves larger number of the slip systems (1/2〈1 1 1〉{1 1 0}, 1/2〈1 1 1〉{1 1 2}, 1/2〈1 1 1〉{1 0 1}) than it takes place at deformation of the surface (0 0 1) (1/2〈1 1 1〉{1 1 2}, 1/2〈1 1 1〉{1 0 1} [16]), and this leads to different degrees of their hardening.

The observed differences in the character of concentration dependence of microhardness for the pyramidal and prismatic sectors (Fig. 3) is obviously connected with different character of the interaction between the impurity ions and l-arg molecules at high and low indenter loads.

**Fig. 8:**
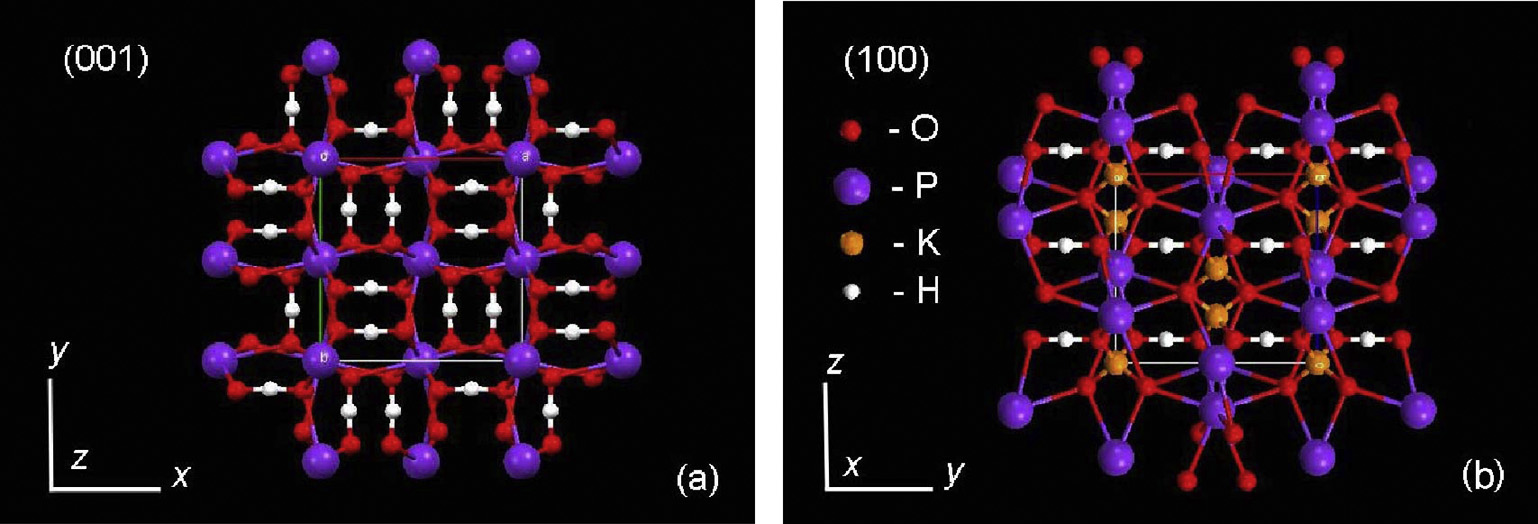
Projection of KDP crystal structure onto the planes (0 0 1) (a) and (1 0 0) (b).

The observed decrease of microhardness values of KDP crystals doped with l-arg in comparison with those of pure KDP seems to be bound up with weakening of the interatomic bonds in the crystal lattice due to incorporation of 1.4 wt% of l-arg. As shown in [10], [11], l-arg molecules enter into the prismatic and pyramidal growth sectors of KDP crystal that is caused by the ability of the amino acid molecules to form hydrogen bonds with the growing crystal face {1 0 0}, and to interact electro statically with the positively charged face {1 0 1}. Presented in Fig. 8 are the projections of the structure of KDP crystal on the planes (0 0 1) and (1 0 0). The crystal lattice consists of the phosphate groups PO_4_^3−^ linked by the directed hydrogen bonds O— H⋯O parallel to the directions [1 0 0] and [0 1 0] (in Fig. 8a they are shown by white lines). Located between the tetrahedrons are potassium ions, each of the latter is surrounded by 8 oxygen atoms which belong to the tetrahedrons PO_4_^3−^. The ionic bonds К—O are of two types – practically parallel and practically perpendicular to the direction [0 0 1] (Fig. 8b).

Doping of KDP crystal with the amino acid increases the lattice parameter *a* in comparison with the one of pure KDP by 2.45 · 10^−4^ Å and 2.42 · 10^−4^ Å, respectively, for the samples cut out from the prismatic and pyramidal sectors. Thereat, the parameter *c* of the doped crystal diminishes with respect to that of the pure crystal: Δ*c* = −8.0 · 10^−5^ Å (the prismatic sector) and Δ*c* = −9.5 · 10^−5^ Å (the pyramidal sector) [11]. Thus, both growth sectors show “extension” of the elementary cell along the direction *a* and “compression” along the direction *c* (Fig. 8) which decrease the lattice energy due to transformations in the system of hydrogen bonds and changes in electrostatical interaction between the ions К—O.

KDP crystals pure and doped with l-arg are characterized by high propensity to brittle fracture (Table 1). The fracture toughness of these crystals is of the same level as in the optical materials such as the crystals of lithium-gadolinium borate – 0.41 MPa·m^1/2^ and lanthanum metaborate – 0.38 MPa·m^1/2^ [17]. As shown earlier [6], [7], perfect cleavage is not characteristic of KDP crystals, however, they show microcleavage under the indentor parallel to the planes (0 0 1), (1 0 0) and {2 2 1}. The pattern observed for the doped crystals is analogous. Well-developed radial cracks propagate from the corner of the indentations for loads exceeding 20 N. At loads of 0.5–2 N linear plots of *l* against *P* (Fig. 6) have a slope of nearly 2/3, consistent with *l* ∼ *P*^2/3^ [12]. At low loads the slope of these plots is not exactly 2/3, as expected from fracture mechanics concept. The surface stresses caused by mechanical treatment lead to expansion of surface traces of the cracks.

Studies of the bulk laser-induced damage of optical materials have usually been directed towards measuring its threshold, and only few researchers have studied bulk damage morphology [18]. The post-exposure pattern of the damaged surfaces of KDP is similar to the one of crack formation at deformation under concentrated load. Certain disagreement between the obtained values of mechanical and laser damage resistance (Table 1, Table 2) seem to be due to different conditions of the formation of cracks in the crystal caused by concentrated load and laser pulses. The morphology of optical breakdown [19] in the range of nanosecond pulses is represented by melted zone with cracks, since damage is formed under the conditions of local heating at the laser focus point.

## 5 Conclusions

KDP crystals pure and doped with l-arg (0.3, 0.4, 1.0, 1.4 wt% of amino acid in the solution) were grown by the method of temperature reduction. It is shown that at loads of 0.2–2 N pure KDP and KDP:l-arg crystals show “reverse indentation size effect” connected with the influence of the distorted zone of the crystal surface. For the crystals containing 0.3 and 0.4 wt% of l-arg the microhardness values rise. Doping of KDP with 1.4 wt% of l-arg leads to loss of hardness of both faces by ∼5–9 % and ∼14–18 % in the pyramidal and prismatic sectors, respectively. Brittle fracture of the pure and doped crystals has the same regularity, the planes of easy crack propagation are {2 2 1}, (1 0 0) and (0 0 1). The crack resistance of KDP crystals doped with 0.3 and 0.4 wt% of l-arg remains unchanged. The presence of 1.4 wt% of l-arg diminishes the coefficient of stress intensity. The pattern of crack formation in the zone of laser radiation action is similar to the one of brittle fracture obtained at local load application. The plane of predominant crack propagation is {2 2 1}. Strength characteristics of the doped crystals vary insignificantly all over the range of the investigated concentrations of l-arg. Thus doping with amino acid does not promote cracking under mechanical or thermal exposure in the process of generation of higher harmonics.

## References

[1] Lu G.W., Sun X. (2002). Raman study of lattice vibration modes and growth mechanism of KDP single crystals. Cryst. Res. Technol..

[2] Pritula I.M., Kolybayeva M.I., Salo V.I., Puzikov V.M. (2007). Defects of large-size KDP single crystals and their influence on degradation of the optical properties. Opt. Mater..

[3] Parikh K.D., Dave D.J., Parekh B.B., Joshi M.J. (2007). Thermal, FT–IR and SHG efficiency studies of l-arginine doped KDP crystals. Bull. Mater. Sci..

[4] Saravanan R.R., Seshadri S., Murugan M., Manivannan V. (2013). Structural, optical properties and effect of amino acid on growth of KDP crystals. Indian J. Pure Appl. Phys..

[5] Dave D.J., Parekh K.D., Joshi M.J. (2013). Vickers micro-hardness studies of amino acids (l-histidine, l-threonine and dl-methoinine) doped KDP crystals. Adv. Mater. Res..

[6] Atroschenko L.V. (1987). Influence of microbrittleness on the character of laser damage in KH_2_PO_4_ single crystals. Phys. Chem. Mater. Treat..

[7] L.V. Atroschenko, Microbrittleness anisotropy of KH2PO4 single crystals, in: Institute for Single Crystals, Kharkov (Ed.), Obtaining and Investigation of Optical and Scintillation Materials, vol. 12, 1984, pp. 30–33 (in Russian).

[8] Fang T., Lambropoulos J.C. (2002). Mcrohardness and indentation fracture of potassium dihydrogen phosphate (KDP). J. Amer. Ceram. Soc..

[9] Xu D., Xue D., Ratajczak H. (2005). Morphology and structure studies of KDP and ADP crystallites in the water and ethanol solutions. J. Mol. Struct..

[10] Kostenyukova E.I., Bezkrovnaya O.N., Tkachenko V.F. (2015). Effect of l-arginine on the optical properties, crystalline perfection and laser damage threshold of KDP crystals. Funct. Mater..

[11] Pritula I.M., Kostenyukova E.I., Bezkrovnaya O.N., Kolybaeva M.I. (2016). KDP crystal doped with l-arginine amino acid: growth, structure perfection, optical and strength characteristics. Opt. Mater..

[12] Anstis G.R., Chantikul P., Lawn B.R., Marshall D.B. (1981). Critical evaluation of indentation techniques for measuring fracture toughness: I, direct crack measurements. J. Am. Ceram. Soc..

[13] Sangwal K. (2000). On the reverse indentation size effect and microhardness measurement of solids. Mater. Chem. Phys..

[14] Pritula I.M., Velikhov Yu.N. (1999). Some aspects of UV-absorption of NLO KDP crystals. Proc. SPIE: Int. Soc. Opt. Eng..

[15] de Vries S.A., Goedtkindt P., Bennett S.L. (1998). Surface atomic structure of KDP crystals in aqueous solution: an explanation of the growth shape. Phys. Rev. Lett..

[16] Guin C.H., Katrich M.D., Savinkov A.I., Shaskolskaya M.P. (1980). Plastic strain and dislocation structure of the KDP group crystals. Kristall und Technik..

[17] Dolzhenkova E.F., Baumer V.N., Gordeev S.I. (2003). Fracture toughness and crystallographic characteristics of Li_6_GdB_3_O_9_ single crystals. Crystallogr. Rep..

[18] Salo V.L., Atroshenko L.V., Garnov S.V., Khodeyeva N.V. (1996). Structure, impurity composition, and damage threshold of the subsurface layers in KDP and DKDP single crystals. Proc. SPIE: Int. Soc. Opt. Eng..

[19] Yoshida H., Jitsuno T., Fujita H. (2000). Investigation of bulk laser damage in KDP crystal as a function of laser irradiation direction, polarization, and wavelength. Appl. Phys. B.

